# Rapid recruitment of large cohort to support trials in general practice: the role of FARSITE

**DOI:** 10.1186/1745-6215-16-S2-O75

**Published:** 2015-11-16

**Authors:** Peter Bower, Kelly Howells, Sheila McCorkindale, Lucy Bridges, Mark Sidaway

**Affiliations:** 1 University of Manchester, Manchester, UK; 2 Clinical Commissioning Group Salford, Salford, UK; 3 NorthWest EHealth, Manchester, UK; 4 Salford Royal Foundation Trust, Salford, UK

## Introduction

There is increasing interest in the cohort multiple randomised controlled trial (cmRCT - Relton BMJ doi: 10.1136/bmj.c1066) as a model for pragmatic trials in general practice. We have adopted the design for our CLASSIC study of integrated care (http://www.nets.nihr.ac.uk/projects/hsdr/1213033). However, the cmRCT needs rapid recruitment of large patient cohorts, which can be a logistical challenge.

Traditionally, general practices must make the initial approach to patients to take part in trials. This requires investment of time and resources, acting as a barrier to GPs who might be interested in research.

## Methods

CLASSIC employed an innovative approach using FARSITE, rapid search software which allows researchers to search anonymised health records, while ensuring GP control over recruitment (http://nweh.org.uk/products/farsite).

FARSITE was used to manage CLASSIC recruitment centrally, sending lists of eligible patients to 33 GP practices within Salford. Once approved by GPs, a link with a third-party service enabled 12,000 invitation letters to be mailed remotely. This saved individual practices from the burden of managing this process.

## Results

In total, 4,300 patients were recruited over 4 months. By reducing workload and disruption, FARSITE enabled us to conduct CLASSIC more efficiently, to ensure participation across almost all local practices in the area, maximising coverage and equitable access for older patients to NIHR research.

## Conclusion

Encouraging greater engagement in research is a key priority for Greater Manchester and Salford, and all participating practices now have access to FARSITE Recruitment.

